# Expression patterns and clinical implications of PDL1 and DLL3 biomarkers in small cell lung cancer retrospectively studied: Insights for therapeutic strategies and survival prediction

**DOI:** 10.1016/j.heliyon.2024.e27208

**Published:** 2024-02-29

**Authors:** Kalliopi Domvri, Alexey V. Yaremenko, Apostolos Apostolopoulos, Savvas Petanidis, Sofia Karachrysafi, Nikoleta Pastelli, Theodora Papamitsou, Styliani Papaemmanouil, Sofia Lampaki, Konstantinos Porpodis

**Affiliations:** a Laboratory of Histology-Embryology, School of Medicine, Aristotle University of Thessaloniki, Thessaloniki, Greece; b Pathology Department, George Papanikolaou Hospital, Aristotle University of Thessaloniki, Thessaloniki, Greece; c Brigham and Women's Hospital, Harvard Medical School, Boston, MA, USA; d Pulmonary Department, Oncology Unit, George Papanikolaou Hospital, School of MedicineAristotle University of Thessaloniki, Thessaloniki, Greece

**Keywords:** SCLC, Biomarkers, PDL1, DLL3, Histopathology, Biopsy

## Abstract

Lung cancer is a leading cause of cancer-related deaths globally, includes small cell lung cancer (SCLC), characterized by its aggressive nature and advanced disease at diagnosis. However, the identification of reliable biomarkers for SCLC has proven challenging, as no consistent predictive biomarker has been established. Nonetheless, certain tumor-associated antigens, including programmed death-ligand 1 (PDL1) and Delta-Like Ligand 3 (DLL3), show promise for targeted antibody-based immunotherapy. To ensure optimal patient selection, it remains crucial to comprehend the relationship between PDL1 and DLL3 expression and clinicopathological characteristics in SCLC. In this study, we investigated the expression patterns of PDL1 and DLL3 biomarkers in endobronchial samples from 44 SCLC patients, examining their association with clinical characteristics and survival. High PDL1 expression (>1%) was observed in 14% of patients, while the majority the SCLC patients (73%) exhibited high DLL3 expression (>75%). Notably, we found a positive correlation between high PDL1 expression (>1%) and overall survival. However, we did not observe any significant differences in the biomarkers expression concerning age, sex, disease status, smoking status, or distant metastases. Further subgroup analysis revealed that a high co-expression of both PDL1 (>1%) and DLL3 (100%) antigens was associated with improved overall survival. This suggests that SCLC expressing PDL1 and DLL3 antigens may exhibit increased sensitivity to therapy, indicating their potential as therapeutic targets. Thus, our findings provide novel insights into the simultaneous evaluation of PDL1 and DLL3 biomarkers in SCLC patients. These insights have significant clinical implications for therapeutic strategies, survival prediction, and development of combination immunotherapies.

## 1 Introduction

Lung cancer remains a prominent contributor to cancer-related deaths on a global scale. Among the various types of lung cancer, small cell lung cancer (SCLC) accounts for approximately 15% of cases and is characterized by its highly aggressive nature, often resulting in extensive disease at the time of diagnosis [1,2].

The identification of reliable biomarkers in SCLC has been challenging, and until recently, no consistent predictive biomarker of response had been identified. Tumor suppressor genes, tumor protein p53 and retinoblastoma protein 1 (RB1), are found in nearly all cases of SCLC but are not currently targetable due to poor genomic stability. However, the presence of tumor-associated antigens, which can potentially be targeted with immunotherapy, persists [3,4]. One such biomarker is Delta-Like Ligand 3 (DLL3), a suppressor of NOTCH, known to be highly expressed on the surface of SCLC cells. Patients with DLL3-positive circulating tumor cells have shown decreased overall survival (OS) and progression-free survival (PFS) compared to DLL3-negative patients. Consequently, DLL3-targeted investigational products are now being investigated in clinical trials [5,6]. While DLL3 expression from SCLC biopsies is often limited and impractical, immunohistochemistry is commonly used to predict the effects of DLL3-targeted agents [7].

In addition to DLL3, the tumor mutational burden (TMB), characterized by a high number of somatic non-synonymous mutations, has recently emerged as a potential biomarker for SCLC patients [8]. However, the specific TMB cutoff for patient selection in SCLC has yet to be clearly established.

The role of programmed death-ligand 1 (PDL1) expression in SCLC patients has also been under investigation, but the results have been inconsistent. The IMpower 133 study evaluated the safety and efficacy of Atezolizumab, an anti-PDL1 monoclonal antibody, in combination with standard first-line chemotherapy compared to placebo. This study identified Atezolizumab as the first agent to demonstrate a survival benefit in treatment-naïve metastatic SCLC patients after three decades [9]. Subsequently, further studies have been designed to investigate the potential benefits of immunotherapy as a first-line treatment for SCLC [10]. Other anti-PD1 monoclonal antibodies, such as nivolumab and pembrolizumab, have been approved for third-line treatment of SCLC [11]. However, the identification of reliable biomarkers that can identify subsets of patients who may benefit from immunotherapy remains crucial. On this concept a recent study reported that silencing the transducer and transmembrane-signal regulator (GNG12) gene leads to the downregulation of the PD-L1 gene transcription, thus, GNG12 can be suggested as a cancer risk factor and can be considered as a possible immunotherapy target [12].

Furthermore, ongoing investigations are exploring the combination of immune checkpoint inhibitors (ICIs) with other treatment modalities in SCLC [10]. However, the reasons behind which subset of SCLC patients may benefit from ICIs and their potential efficacy when combined with targeted therapies or cytotoxic agents are still unknown. Therefore, understanding the association between PDL1 and DLL3 expression and the clinicopathological features of SCLC is of paramount importance.

In our study, we aimed to investigate the expression patterns of PDL1 and DLL3 in endobronchial samples from SCLC patients and examine their associations with various clinical characteristics including age, sex, disease status, presence of metastasis, smoking status, and survival. We specifically focused on determining the correlation between high PDL1 expression levels and the overall survival of SCLC patients. Additionally, we explored the association between the co-expression of PDL1 and DLL3 and clinical characteristics, as well as prognostic features, and observed that high co-expression of these antigens correlated with increased overall survival in patients. Importantly, to our knowledge, this is the first study to simultaneously investigate both PDL1 and DLL3 biomarkers in endobronchial biopsies while exploring their correlation with clinical characteristics and overall survival in SCLC patients. The results obtained from our study have significant relevance for the development of novel approaches aimed at determining SCLC's sensitivity to anticancer therapy, predicting overall survival, and designing rational combination cancer immunotherapies targeting PDL1 and DLL3 antigens.

## 2 Materials and methods

### 2.1 Patients and endobronchial samples

This is a retrospective study, approved by the Scientific and Ethical Committee of George Papanikolaou Hospital (Approval No. 85_13/JAN/2021). The inclusion criteria for patients were based on histological confirmation of small cell lung cancer (SCLC) as per the classification provided by the World Health Organization in 2004 [13]. The disease status of the patients was determined using the 7th edition of the TNM staging system [14].

A total of forty-four endobronchial biopsies from SCLC patients were randomly selected in January 2021 to ensure a representative sample and generalizability. All patients included in the study had unfortunately passed away and had been treated at George Papanikolaou Hospital between 2014 and 2018. As part of their treatment, all patients had received platinum-based chemotherapy as a first-line treatment, and half of the patients had also undergone radiotherapy, in accordance with the ESMO Clinical Practice Guidelines [15].

The endobronchial samples from the patients were collected at the time of diagnosis, fixed in formalin, and then embedded in paraffin. These samples were subsequently stored in the Pathology Department of George Papanikolaou Hospital until they were selected for laboratory assessment.

### 2.2 Immunohistochemistry analysis

The assessment of PDL1 and DLL3 expression involved analyzing 5-μm sections derived from formalin-fixed and then paraffin-embedded SCLC cancer tissue blocks using an anti-PDL1 antibody (clone IHC411, GenomeMe, Richmond, Canada) on the LEICA BOND-III platform and anti-DLL3 antibody (clone SP347, Spring Bioscience, Pleasanton, CA) on VENTANA platform.

Stained slides were then observed under a light microscope (Nikon, ECLIPSE 50i microscope) to assess positivity, attached to an image analysis system (Nikon Digital sight DS-L2, Nikon Corporation, Japan) for recording images. Two pathologists blindly calculated both biomarkers for interobserver and intraobserver variability. Positivity of DLL3 expression was evaluated at 4× magnification, with any membranous or cytoplasmic staining observed in total tumor cells, as it was outlined previously [16]. DLL3-high was designated when 75% or more of the tumor cells were stained, consistent with the criteria applied in the Rova-T clinical trial, while DLL3-low was characterized by less than 75% positively stained cells. The PD-L1 tumor proportion score (TPS) was determined by calculating the percentage of at least 100 viable cancer cells exhibiting partial or complete staining of the membranes. During the examinations, necrotic areas were not analyzed and scored. PDL1 positivity and PDL1 high expression were characterized as staining in ≥1% of total cancer cells, as used in other clinical trials (membranous >1 %) [17]. No differences were reported among the calculations of the two pathologists.

### 2.3 Statistical analysis

The sample size was determined using the G*Power software (Die Heinrich-Heine-Universität Düsseldorf, Germany) for a two-sided test, with a power of 0.95 and a statistical significance level of 0.05. An effect size of 0.5 was calculated, resulting in a total sample size of 42 participants. For the performing of statistical analysis, SPSS was utilized (version 21.0 IBM-SPSS statistical software, Armonk, NY, USA). Descriptive statistics were used, with quantitative data presented as means with standard deviation (SD) or medians with minimum-maximum range. Normality tests using the Shapiro–Wilk test were performed to differentiate parametric from non-parametric variables. Qualitative variables were summarized as frequencies and percentages. Group differences were assessed using two-tailed Student's t-tests or ANOVA in cases with parametric variables. Kruskal–Wallis or Mann–Whitney U tests were performed for non-parametric variables. Survival analyses were conducted using Kaplan-Meier curves with log-rank tests. Statistical significance was set at p < 0.05.

## 3 Results

### 3.1 General clinicopathological characteristics of SCLC patients included in the study

In this research, we aimed to investigate the association between PDL1 and DLL3 expression patterns and the clinicopathological features of patients with small cell lung cancer (SCLC). A total of forty-four endobronchial biopsies from SCLC patients were randomly selected for inclusion in this study. To provide an overall picture of the clinical characteristics of the selected SCLC patients, we evaluated various parameters (Table 1). The study population consisted of 44 SCLC patients with a median age of 65 ± 8 years old and a performance status of 0–2. These patients were treated with either first-line platinum-based chemotherapy or a combination of first-line platinum-based chemotherapy and radiotherapy. The majority of the patients were male (86%), and all patients had a history of smoking, with 61% being current smokers and 39% being ex-smokers. At the time of diagnosis, a significant proportion of patients (68%) had extensive disease, and 70% of patients had metastases in various organs. The median progression-free survival for the patients was 6 months, and the median overall survival for all patients included in the study was 8.5 months. These findings provide important insights into the clinical outcomes of SCLC patients and serve as a foundation for further analysis of PDL1 and DLL3 expression patterns in relation to these clinicopathological features.

**Table 1:** Clinical characteristics of small cell lung cancer patients included in the study.

Characteristics	Patients (n = 40) n (%)
Age, mean (range), years	65 (49–81)
Age >60	27 (61%)
Age ≤60	17 (39%)
Sex, Male/Female	38/6 (86%/14%)
Smoking status-Pack/years, mean ± sd	61 ± 24
Current smoker	27 (61%)
Ex-smoker	17 (39%)
PS	
O	38 (86%)
1	4 (9%)
2	2 (5%)
Disease status (LD/ED)	14/30 (32%/68%)
Brain metastases	5 (11%)
Bone metastases	9 (20%)
Other metastases	17 (39%)
Radiation	22 (50%)
OS, median ± sd, months	8.5 ± 5
PFS, median ± sd, months	6 ± 3

Abbreviations: PS: Performance status, LD: Limited disease, ED: Extensive disease, sd: standard deviation, OS: Overall Survival, PFS: Progression-Free Survival.

### 3.2 PDL1 and DLL3 expression patterns in SCLC patients

**Fig. 1:**
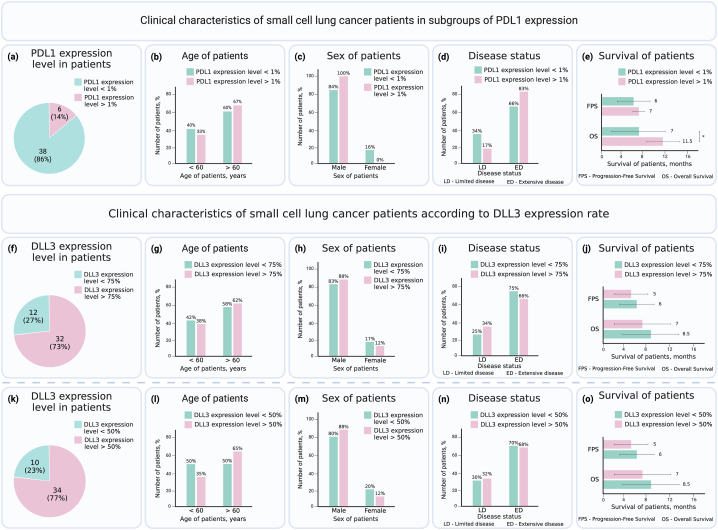
Clinical Characteristics of small cell lung cancer (SCLC) patients (n = 44) in Subgroups of PDL1 expression (a–e) and subgroups of DLL3 expression based on expression levels at the 75th percentile (f–j) and 50th percentile (k–o). Asterisks (*) indicate significant differences determined by Student's t-test, with *p < 0.05.

**Fig. 2:**
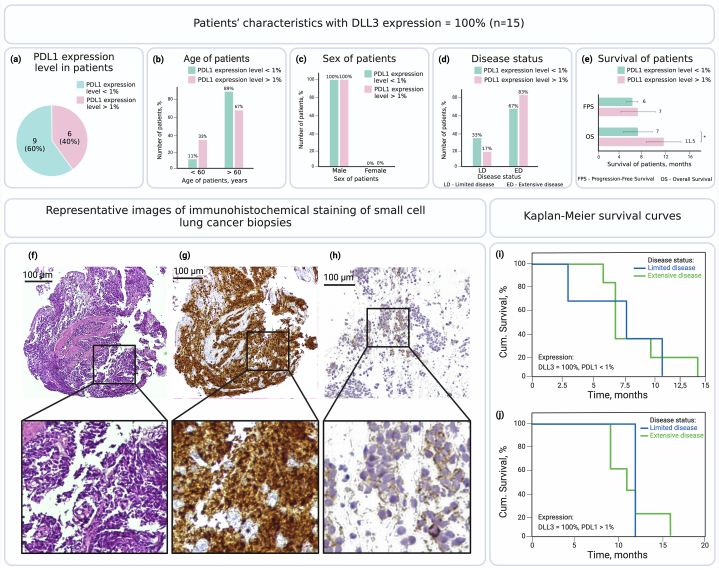
(a–e) Clinical characteristics of small cell lung cancer (SCLC) patients with DLL3 expression = 100% (n = 15); (f–h) Representative images of immunohistochemical staining of SCLC biopsies: (f) Hematoxylin and Eosin (H&E) Stain (×100 magnification) demonstrating the morphology of the small cell lung carcinoma (SCLC), small cells with minimum cytoplasm, intensely colored nuclei, (g) DLL3-high expression on SCLC (×100 magnification) and (h) PDL1> 1% expression on SCLC (×400 magnification); (i–j) Kaplan-Meier survival curves for patients with SCLC with DLL3 expression = 100%: (i) PDL1 < 1% (log-rank p-value: 0.855), and (j) PDL1 > 1% (log-rank p-value: 0.753). Asterisks (*) indicate significant differences determined by Student's t-test, with *p < 0.05.

The expression of tumor-associated antigens PDL1 and DLL3 may be correlated with the clinical characteristics of SCLC patients. To investigate this correlation, we studied the dependence of clinical characteristics on PDL1 and DLL3 expression levels (Fig. 1). Representative immunohistochemical images illustrating PDL1 and DLL3 expression are provided in Fig. 2.

An analisis of clinical characteristics of patients with PDL1 expression showed that 86% of the patients had PDL1 expression levels below 1%, while 14% of the patients had expression levels higher than 1% (Fig. 1a). Following evaluation of the association between clinical characteristics and high or low expression of PDL1 demonstrated, that age, sex, and disease status did not significantly correlate with PDL1 expression levels (Fig. 1b–d). However, we observed that patients with PDL1 expression higher than 1% had a significantly longer overall survival (11.5 ± 3 months) compared to patients with PDL1 expression lower than 1% (7 ± 5 months) (Fig. 1e). These findings suggest that PDL1 expression may potentially serve as a prognostic marker for overall survival in SCLC patients.

Other biomarker SCLC can be DLL3, so next, we investigated the association between DLL3 expression in SCLC patients and clinical features. In our study, 73% of the patients exhibited DLL3 expression levels higher than 75% (Fig. 1f), suggesting a strong association of this antigen with SCLC. However, further analysis did not reveal any significant correlation between DLL3 expression levels and clinical characteristics such as age, sex, disease status, or survival of patients (Fig. 1g–j). To be sure that our assumprion was correct, we decided to analyze additionally subjects with a lower cut-off value of 50% for DLL3 expression. By the way, this adjustment yielded a similar group size, and no significant differences in clinical characteristics were found between the two groups (Fig. 1f-o). These findings suggest that DLL3 expression alone may not be predictive of specific clinical or prognostic characteristics in our study population.

### 3.3 Prognostic and therapeutic value of PDL1 and DLL3 co-expression in SCLC patients

However, in our study, we observed a subgroup of patients with DLL3 expression level of 100% (n = 15), where all patients with PDL1 expression level higher than 1% expression were included. We then compared the groups of patients with DLL3 expression 100% and PDL1 expression level higher and lower than 1%. In contrast to the general group, 40% of patients in this subgroup had PDL1 expression levels higher than 1% (Fig. 2a), which is more than twice higher than in the general group (14%, n = 44) (Fig. 1a).

While there were no significant differences observed in demographic and clinical characteristics concerning age, sex or disease status (Fig. 2b–d), we noted differences concerning survival of patients. Interestingly, median overall survival was significantly higher (11.5 ± 2.5 months, p = 0.036) in the group of patients with 100% DLL3 expression and PDL1 expression levels higher than 1% when compared to patients with PDL1 expression levels lower than 1% (7 ± 3 months) (Fig. 2e), indicating a distinct subgroup of patients with a favorable tumor profile, possibly more sensitive to treatment. Also, in this subgroup analysis, median overall survival in patients with 100% DLL3 expression and PDL1 expression higher than 1% with the extensive disease (n = 5) was found 12 ± 3 months, whereas in patients with 100% DLL3 expression and PDL1 expression lower than 1% with extensive disease (n = 6) was found 7 ± 3 months. Representative images of Hematoxylin & Eosin staining and immunohistochemical staining of SCLC biopsies are shown in Fig. 2f-h.

Kaplan-Meier survival curves for SCLC patients with 100% DLL3 expression and PDL1 expression lower or higher 1% regarding their disease status appear in Fig. 2(i and j). Concerning progressive-free survival results, no significant differences were observed between high and low expression groups of either PDL1 or DLL3 proteins. Altogether, these results suggest that the evaluation of co-expression of DLL3 and PDL1 antigens may potentially be used for predicting sensitivity of SCLC to treatment and overall survival in SCLC patients.

## 4 Discussion

Despite the initial effectiveness of chemotherapy and radiotherapy in SCLC patients, relapse and tumor resistance commonly occur. Unfortunately, treatment options such as targeted therapies and biomarkers are limited in clinical practice for these patients. Thus, this study aimed to investigate the expression patterns of PDL1 and DLL3 proteins in endobronchial biopsies of SCLC patients.

Our findings demonstrate that DLL3 is highly expressed in SCLC patients, supporting the conclusion that biopsy specimens can be relied upon as a source for evaluating DLL3 in targeted therapy. Additionally, we found that PDL1 expression >1% was observed in 14% of the studied population. Furthermore, we examined the clinical and prognostic significance of these proteins. Notably, our study revealed that patients with an expression pattern of PDL1 >1% and high DLL3 expression (=100%) represent a distinct subpopulation of SCLC patients who may have a more treatment-sensitive tumor. To the best of our knowledge, this is the first study to analyze both PDL1 and DLL3 expression levels in endobronchial biopsies of SCLC patients.

In our study, PDL1 expression >1% was observed in 14% of the population studied. Similarly, according to previous studies, histological examinations of SCLC specimens have demonstrated that <20% express PDL1 expression >1% of tumor cells [8,[18], [19], [20], [21]]. It has been found that there is variation in the frequency of PD-L1 expression in SCLC tumor cells among studies. The conclusions drawn from these studies are limited because of differences in sample size, the specific antibody used, the staining pattern (whether it is membranous or cytoplasmic), and the cut-off values. For example, a study evaluating the efficacy of maintenance pembrolizumab in extensive-stage SCLC patients reported 10% of patients with PDL1 expression ≥1% in tumor cells [18]. However, the association between PDL1 expression and overall survival in SCLC patients receiving chemotherapy in combination with an immune checkpoint inhibitor (ICI) remains inconsistent. According to a recent meta-analysis, which included 27 studies and enrolled a total of 2792 patients, positive PD-L1 expression was found to be a favorable prognostic factor for SCLC, but the statistical significance of this finding was not established [22]. While PDL1 expression has been mostly linked to a higher response rate in other tumors like NSCLC [23], findings from trials such as IMPower 133 suggest that PDL1 expression is not predictive of overall survival in SCLC patients receiving chemotherapy with an ICI [17]. Conversely, studies like KEYNOTE-028 and KEYNOTE-158 show a correlation between PDL1 positivity and benefit from pembrolizumab [24]. Additionally, recent research indicates the presence of tumor-infiltrating lymphocytes in SCLC patients without detectable PDL1 expression [25], suggesting the potential clinical role of alternative immune checkpoints in the tumor microenvironment.

Ongoing and future trials are required to determine whether ICIs alone will be the most successful therapeutic strategy in SCLC or if their combination with novel targeted therapies will offer greater benefits. Therefore, this study also evaluated DLL3 expression in SCLC patients. We observed high DLL3 expression levels in 73% of endobronchial biopsies, using a cutoff rate of 75% based on ongoing rovalpituzumab tesirine (Rova-T) clinical trials [26]. Rova-T is an antibody-drug conjugate that targets DLL3 and delivers a cytotoxin into tumor cells. Similar findings of high DLL3 expression in SCLC patients have been reported in previous studies [[27], [28], [29]]. Furthermore, using a cutoff rate of 50%, we observed DLL3 high expression levels >50% in 77% of our population. These findings align with phase I of the Rova-T clinical trial [16] and a study by Furuta et al. [30], which reported DLL3 high expression rates of 67% and 47%, respectively.

Regarding the association between DLL3 expression and clinical characteristics, no significant correlation was found in our study, consistent with a previous report [29]. However, other studies have associated high DLL3 expression with the smoking history of patients and higher prevalence in patients with lymph node metastasis [30].

In terms of survival, DLL3 expression was not found to be related to the survival of our patients, which is consistent with a study by Tanaka et al. [27]. However, Regzedmaa et al. reported that SCLC patients with high DLL3 expression had poorer outcomes [29]. Disappointingly, a phase III clinical trial that aimed to evaluate the effectiveness of Rova-T versus topotecan as a second-line treatment for advanced SCLC patients with high levels of DLL3 was halted prematurely due to a reduction in overall survival in the Rova-T arm [31]. Despite the inconsistent results and the toxicity profile observed with anti-DLL3 treatment, DLL3 remains a promising target in SCLC, as it is homogeneously expressed on the surface of tumor cells in approximately 69% of SCLC cases and not in normal tissue [32,33]. Despite the toxicity profile of rovalpituzumab tesirine observed and the inconsistent results of anti-DLL3 treatment [16], DLL3 remains a promising target in SCLC.

Although our study suggests that DLL3 may have a relatively small impact on the survival of SCLC patients based on our data, interestingly, patients with 100% DLL3-high expression and PDL1 expression >1% seem to have better survival. Therefore, we assume that these patients might derive even greater benefit from combination treatment with anti-PDL1 and anti-DLL3. Despite the small sample size, statistical significance was reached. Furthermore, according to preclinical models, administering sub-efficacious doses of Rova-T resulted in an antitumor response that boosted the effectiveness of immunotherapies in an experimental model of SCLC [34]. The researchers specifically proposed that combining Rova-T with anti-PD(L)1 could be advantageous for patients by activating multiple pathways synergistically, thus enhancing the effects of immune system targeting and directly attacking SCLC tumor cells that express DLL3. Ongoing studies investigating treatment combinations of Rova-T with immunotherapy will provide further insights into the potential of this novel approach in SCLC patients.

It is important to acknowledge that this is a single-center study with a retrospective design and some limitations, including a small sample size and inclusion of immunotherapy or targeted treatment-naïve patients only. However, the observed PDL1 expression values in our population align with previous reports, and DLL3 expression is indeed highly expressed in SCLC patients. Despite the variability in DLL3 expression cutoff values, DLL3 remains a predictive biomarker for the therapeutic utility of Rova-T. Furthermore, pathologists can reliably evaluate DLL3 expression in SCLC biopsies. However, sampling bias resulting from intratumoral and intertumoral heterogeneity, as well as the limited amount of tissue available from SCLC patients, may reduce the value of DLL3 as a reliable biomarker for targeted therapy. Consequently, researchers have developed an antibody as a real-time, noninvasive, and quantitative approach to facilitate the selection of patients for Rova-T treatment based on in vivo DLL3 expression status using PET imaging in a preclinical mouse model of SCLC [7].

## 5 Conclusions

In this study, we investigated the expression patterns of PDL1 and DLL3 in SCLC patients and assessed their associations with clinical characteristics and prognostic features. Our findings revealed a positive correlation between PDL1 expression level greater than 1% and increased overall survival in SCLC patients. Although the prognostic value of DLL3 protein was not determined, we observed a high expression of DLL3 in 73% of SCLC patients, with 75% expressing it at a significant level. Notably, further investigations into the co-expression of PDL1 and DLL3 demonstrated that patients with high expression levels of both antigens exhibited significantly longer overall survival. Therefore, the co-expression of PDL1 and DLL3 could serve as a valuable clinical parameter for assessing SCLC sensitivity to therapy, predicting overall survival, and may be beneficial in combination treatment approaches.

The emergence of a personalized approach in SCLC shows promise for improving outcomes and providing more appropriate and effective therapies for patients. A better understanding of the immune microenvironment is a crucial area of unmet need in SCLC immunobiology. We believe that a deeper understanding of SCLC biomarkers can lead to the development of optimal treatment strategies and sequencing, ultimately advancing therapeutic outcomes for SCLC patients.

## Funding

The research has received co-financing from the 10.13039/501100000780European Union (European Social Fund- ESF) and Greece through the Operational Programme "Human Resources Development, Education and Lifelong Learning" as part of the project "Reinforcement of Postdoctoral Researchers - 2nd Cycle" (MIS-5033021). The State Scholarships Foundation (ΙΚΥ) has implemented the project.

## Institutional review board statement

The study was carried out in compliance with the principles outlined in the Declaration of Helsinki guidelines, and was approved by the Scientific and Ethical Committee of George Papanikolaou Hospital (85_13/JAN/2021).

## Informed consent statement

Patient consent was waived due to the retrospective nature of the study. Written informed consent was obtained from patients’ relatives.

## Data availability statement

The datasets used and/or analyzed during the present study are available from the corresponding author upon reasonable request.

## CRediT authorship contribution statement

**Kalliopi Domvri:** Writing – review & editing, Writing – original draft, Supervision, Resources, Project administration, Methodology, Investigation, Funding acquisition, Formal analysis, Data curation, Conceptualization. **Alexey V. Yaremenko:** Writing – review & editing, Writing – original draft, Visualization, Investigation, Formal analysis, Data curation. **Apostolos Apostolopoulos:** Formal analysis, Data curation. **Savvas Petanidis:** Methodology, Formal analysis, Data curation. **Sofia Karachrysafi:** Writing – review & editing. **Nikoleta Pastelli:** Methodology, Formal analysis. **Theodora Papamitsou:** Writing – review & editing. **Styliani Papaemmanouil:** Writing – review & editing, Methodology, Formal analysis. **Sofia Lampaki:** Writing – review & editing. **Konstantinos Porpodis:** Writing – review & editing, Supervision.

## Declaration of competing interest

The authors have no competing interests to declare.
